# Bipedal robotic walking control derived from analysis of human locomotion

**DOI:** 10.1007/s00422-018-0750-5

**Published:** 2018-02-05

**Authors:** Lin Meng, Catherine A. Macleod, Bernd Porr, Henrik Gollee

**Affiliations:** 1 Division of Biomedical Engineering, School of Engineering, University of Glasgow, Glasgow, G12 8QQ UK; 2 Department of Biomedical Engineering, University of Strathclyde, Glasgow, G4 0NW UK

**Keywords:** Reflexive rhythmic generator, Robotics, Bipedal locomotion, Limit cycle walking, Biological inspiration, Human walking

## Abstract

This paper proposes the design of a bipedal robotic controller where the function between the sensory input and motor output is treated as a black box derived from human data. In order to achieve this, we investigated the causal relationship between ground contact information from the feet and leg muscle activity n human walking and calculated filter functions which transform sensory signals to motor actions. A minimal, nonlinear, and robust control system was created and subsequently analysed by applying it to our bipedal robot RunBot III without any central pattern generators or precise trajectory control. The results demonstrate that our controller can generate stable robotic walking. This indicates that complex locomotion patterns can result from a simple model based on reflexes and supports the premise that human-derived control strategies have potential applications in robotics or assistive devices.

**Electronic supplementary material:**

The online version of this article (10.1007/s00422-018-0750-5) contains supplementary material, which is available to authorized users.

## Introduction

Human walking is an inherently complicated task requiring the coordination of several degrees of freedom coupled with highly nonlinear dynamics (Inman et al. 1981). Locomotion arises through the interaction of neural activity and the biomechanical body with the environment. The human must be understood as an integrated system, particularly in motor control, where the behavioural consequences of neural activity depend on the muscle properties, limb geometry and mechanics. In this paper, we argue that the existence of these biomechanical constraints can be exploited to simplify the problem of locomotion control. This hypothesis was tested through the development of a multi-joint bipedal robotic walker (named RunBot III) with a simple mechanical design and a minimal reflexive controller.

The central nervous system (CNS) is responsible for generating, coordinating and adjusting the motor output to suit the walking environment (Nielsen 2003). This system can be divided into two levels, where the top level is the brain and the low level is in the spinal cord. The brain control can be seen as being task-based, having minimal degrees of freedom, while the flexibility of the muscle activity is driven by local circuits in the spinal cord (Bernstein 1967). At the spinal level, the direct motor responses, in the form of local or monosynaptic reflexes, are elicited by afferent signals from the skin, tendons, and muscles. The spinal circuits can produce reproducible and stable gaits and are known to play a dominant role in invertebrates and vertebrates (Brown 1911; Lundberg 1979; Grillner 1985; Fedirchuk et al. 1998; Rossignol 2000). These biological neural networks in the spinal level are often referred to as central pattern generators (CPGs). However, in humans, CPGs appear to be less crucial for walking and have not been conclusively identified in adult studies (Eidelberg et al. 1981; Hultborn and Nielsen 2007). This finding may indicate that human walking has a higher dependence on intact peripheral feedback and supraspinal control compared to other species. Reflexes have been found to not only contribute to the timing of the stepping but also to the adaptation of the gait pattern and reaction to perturbation. Task- and phase-dependent cutaneous reflexes contribute significantly to the response mechanism in reaction to sudden disturbances (Eng et al. 1994; Zehr et al. 1997) and stabilisation (Rossignol et al. 2005). Load-dependent reflexes play an essential role in regulating the timing of the gait cycle (Dietz and Duysens 2000; Sinkjær et al. 2000) and muscle activations during locomotion, especially in the stance phase (Akazawa et al. 1982). However, overall the debate over the extent of neuronal control in human walking is still ongoing and is unlikely to be resolved in the near future.

Human walking has been extensively studied in the field of biomechanics, often through the measurement of kinematics and kinetics, electromygraphic (EMG) activity (Sutherland 2001; Rose et al. 2006), ground reaction forces (Allard et al. 1998; Bamberg et al. 2008; Whittle 2014), and energy expenditure. Over the decades, the assessment of human gait has yielded a tremendous amount of information. What has been concluded is that the role of the biomechanics of the musculoskeletal system is an inherent part of the human control system. Bernstein (1967) stated that “The coordination of movement is the process of mastering redundant degrees of freedom of the moving organ, in other words, its conversion to a controllable system.” The biomechanical patterns observed over the stride period remain relatively consistent, regardless of the walking speed (Winter 1983a). This is supportive evidence for CNS locomotion control but also suggests that in human locomotion, the role of peripheral feedback is essential in maintaining the phasic relationship of the motor patterns.

Biomechanical patterns are often used to validate theories or identify the strategies employed by the CNS. For instance, studies of mechanical perturbations during walking have been undertaken in order to investigate the role of reflexes in locomotion (Akazawa et al. 1982; Capaday and Stein 1986; Yang et al. 1991; Kearney et al. 1999). EMG signals measured during the gait cycle can be viewed as the resulting motor output of what has been programmed in the CNS (Sherrington 1916). At the kinematic level, the EMG patterns are also a function of the gait kinematics (Grillner 1985). The relationships between gait kinematics and EMG patterns have previously been estimated by muscle-based simulations of dynamic walking (Zajac et al. 2002, 2003). Moving further, a better comprehension of the relationships which exist between the neuro-musculo-skeletal system would significantly advance the understanding of locomotor control.

Classical control approaches employed in bipedal robotics aim to realise dynamic walking by generating a physically feasible motion based on a simple biped model with precise joint-angle or trajectory-based control, including centre of mass (CoM) (Kato et al. 1974), zero moment position (ZMP) (Vukobratović 1973) or virtual model based methods (Pratt et al. 2001). This control strategy has been impressively applied to a series of humanoid robots, such as the well-published bipedal walker ASIMO (Sakagami et al. 2002). However, the gait is less efficient compared to human walking due to high gains required for precision control in the actuators and frequency response of these systems.

A potential solution to this issue came with the advent of dynamic walkers. (McGeer 1990) initially showed that a purely passive dynamic walker with simple mechanics is capable of stable walking. Studies have demonstrated that complex locomotion control can be simplified with the introduction of an appropriate mechanical design (Collins and Ruina 2005; Wisse and Van Frankenhuyzen 2006; Geng et al. 2006; Iida et al 2008). Local oscillators, such as central pattern generators (CPGs) with limited sensory feedback, have been successfully used in a range of dynamic walkers (Collins and Ruina 2005; Wisse 2005; Wisse and Van Frankenhuyzen 2006; Iida et al 2008). However, as a biologically inspired approach, the existence of CPGs is not conclusively described in human walking control. This has promoted the development of locomotion controllers based on reflexes rather than on CPGs (Geyer et al. 2003; Geng et al. 2006). The original RunBot, developed by Geng et al. (2006), was the first dynamic walker exclusively controlled by a purely reflexive controller. RunBot attempted a biologically inspired approach where the sensory signals were translated into motor signals with the help of a neural network incorporating neuronal processing without using precise trajectories or CPG control (Geng et al. 2006; Manoonpong et al. 2007). However, this strategy has limitations in providing a model of the human nervous system, which has significant complexity with numerous unknown variables and the exact functions of neural networks are speculative.

Rather than extending the complexity of previous neural control systems for “biologically inspired” robotic walking, we propose the opposite strategy of a relatively simplistic and novel abstract controller based on actual human walking data. Human walking can be regarded as a generalised control system (Duysens et al. 2002). To create a minimalistic closed-loop system, only knowledge of the causal relationship between foot contact information and the motor activation, which was taken as the muscle activation (EMG), was necessary for our study. We took a black box approach to modelling the CNS during walking and studied how the sensory inputs could be translated into functional motor outputs. The calculation of transfer functions relating sensory information and muscle EMG has been discussed in detail in our previous study (Macleod et al. 2014) where the transfer functions were applied to a prior generation of the RunBot (RunBot II) as a proof of concept. The stable gait cycle generated indicated that the approach had potential for use in robotic control. The present paper extends the work and here we would like to propose a novel model of sensorimotor control based on reflexes where the addition of ankle control is considered. To guide our modelling, we conducted a human data collection study involving ten healthy participants. The human data provided quantifiable information about the coupling between sensory input and motor output to support the design of the model. To demonstrate the feasibility of using this mechanism for robot control, we implemented our model to control the walking motion of a specifically developed biped walker—RunBot III.

**Fig. 1:**
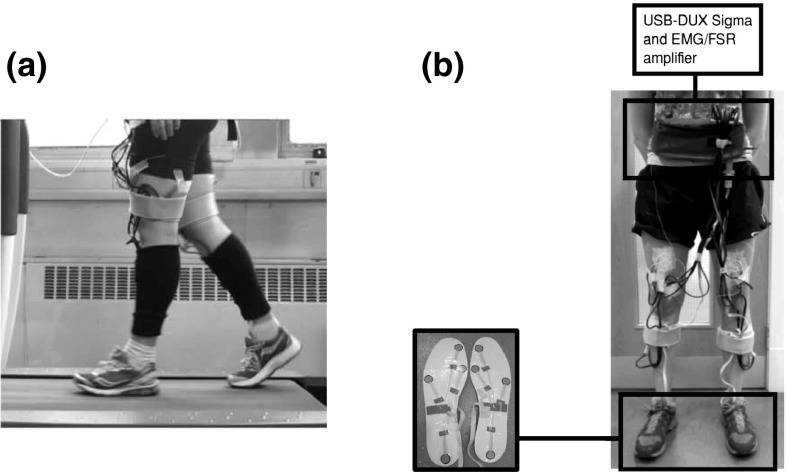
**a** Subject walking on the treadmill during a data collection trial. **b** setup for the treadmill walking trials. The USB-DUX Sigma data acquisition device and EMG/FSR amplifier were worn in a bag around the subject’s waist. Surface EMG electrodes were used to record the muscle activity during the treadmill walking. FSR insoles were placed in the subject’s shoes and measured contact signals under different areas of the feet

The paper is structured as follows: Sect. 2 describes the human walking data collection procedure and data analysis. Section 3 details our robotic model derived from the human data. In Sect. 4 we present the results of our robotic experiments and finally, Sect. 5 is devoted to a discussion of the findings.

## Human walking study

### Ethics statement and walking data collection

The human walking data collection study was granted ethical approval by the University of Strathclyde ethics committee. Ten subjects, four males and six females, with a mean age of 26.5 years (range 23–30 years) were recruited at the Department of Biomedical Engineering, University of Strathclyde and provided informed written consent before taking part.

The data collection comprised of measuring leg muscle EMG and foot contact information during treadmill walking using the setup shown in Fig. 1. EMG from four muscles in both legs was recorded simultaneously during walking. These muscles were chosen due to their different roles in a gait cycle: two muscles [tibialis anterior (TA) and lateral gastrocnemius (LG)] in the shank and two [biceps femoris (BF) and rectus femoris (RF)] in the thigh. Force-sensing resistors (FSRs) (Interlink Electronics, CA, USA) were embedded in standard shoe insoles at four different positions under the feet (toe, 1st metatarsal, 5th metatarsal, and heel) to record foot contact information. All data were recorded with a sampling frequency of 1 kHz using a USB-DUX Sigma data acquisition device (Incite Technology Ltd, Stirling, UK). The treadmill speed was automatically varied using a control programme with small increments or decrements in speed between 0.05 and 0.1 m/s. Here the aim was to limit any dependency on the recorded data with the walking speed. The treadmill control programme generated approximately 100 steps from the participant and the complete sequence had a total walking speed range of 0.39 m/s.

### Data analysis

The EMG signals were filtered using a band-pass filter (50–200 Hz), full-wave rectified and low-pass filtered (6 Hz) to obtain the linear envelope of the EMG. The EMG and FSR sequences were then normalised in amplitude to between 0 and 1 and scaled from 0 to 100$\%$ in every stride to eliminate the effect of inter-subject variation in walking speed. To visualise the relationship between the foot contact and EMG, muscle activity recorded over a given period was averaged in relation to the foot contact to produce an event related average, or ERA. The indication that a motor neuron pool has received suppressed or facilitatory synaptic input is given by troughs or peaks in the ERA of the processed EMG (Davidson et al. 2007).

The entire processed EMG signals, $X_m$ (*m* = BF, RF, TA, LG) and FSR signals $F_i$ [*i* = contralateral heel (CH), ipsilateral heel (IH), ipsilateral toe (IT)], were then used to produce an estimated EMG output signal for each muscle $Y_m$ using the least mean squares (LMS) approach through the convolution of the filter impulse response $h_{m, i}$ with the FSR contact signal $F_{i}$.

1 $$\begin{aligned} Y_m = F_i *h_{m,i} \end{aligned}$$

The error signal *E* is calculated as the difference between the measured EMG signal $X_m$ and the estimated EMG signal $Y_m$.

2 $$\begin{aligned} E = X_m - Y_m \end{aligned}$$

The filter coefficients were updated by an optimisation algorithm driven by the error signal. The duration of the filter response was set to the length of two strides.

3 $$\begin{aligned} h_{m,i, p+1} = h_{m,i} + E \cdot F_i \cdot \mu \end{aligned}$$

where $\mu$ is the learning rate of the adaptive filter, which was set to 0.001.

**Fig. 2:**
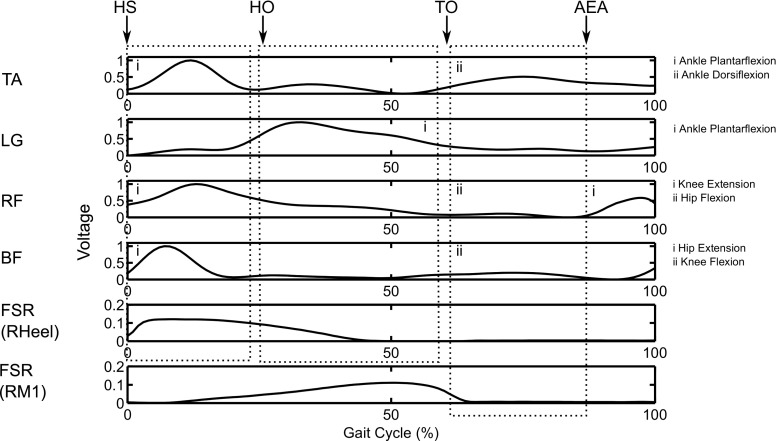
Diagram indicates how the muscle EMG signals are elicited and related to the ground contact information in one gait cycle. The features of the transfer function coefficients are identified corresponding to muscle activity promoting joint movements in human walking. The TA transfer function has two peaks that are responsible for ankle plantarflexion at heel strike (HS) and dorsiflexion after toe off (TO), respectively. The peak in the LG produces ankle push-off and is related to heel off (HO) during walking. The RF transfer function is related to two joint movements: hip flexion at the TO and knee extension when the hip reaches its anterior extreme angle (AEA). The first peak in the BF transfer function is related to hip extension in the stance phase after HS, and the second peak promotes knee flexion in the early swing phase

When given an input of a typical FSR contact signal, the filter produces a muscle activation signal. To compensate for the difference in foot contact sensory feedback between the human and robotic system^1^, the filter coefficients $h_{m, i}$ from the human data were convolved with averaged FSR signals $\overline{F_{i}}$ over two strides:

4 $$\begin{aligned} H_{m, i} = h_{m, i} * \overline{F}_{i} \end{aligned}$$

A half Hanning window was used to extract the filter coefficients for one stride and the amplitude normalised to between 0 and 1. The response of the transfer function, $H_{m, i}$, is therefore equivalent to applying a typical FSR signal measured in human gait, but the RunBot can use an impulse signal to trigger the response. An example of the filter’s output for one stride is shown in Fig. 2, together with the corresponding FSR signals. Further details regarding the human data processing are described in Macleod et al. (2014).

### Muscle functions

A key step was to define the transfer functions related to muscle activation, which in turn promote the biomechanical movements. First these muscle transfer functions need to be translated into joint motions to create an abstract closed-loop control system.

#### Rectus femoris

The RF is a bifunctional muscle responsible for hip flexion in the swing phase and knee extension in the late swing and stance phase. Two peaks are observed in the RF transfer function (Fig. 2). One peak corresponding to the hip flexion relates to the ipsilateral Toe Off (TO). Another peak, which coincides with the late swing, is identified as the muscle activity that is responsible for the knee extension. It is also observed that the knee extension at the late swing phase does not follow any foot contact as it occurs before the ipsilateral Heel Strike (HS). In accordance with the reflexive neuronal controller implemented in the RunBot (Geng et al. 2006), we assumed that the anterior extreme angle (AEA) of the hip activates the RF muscle for knee extension.

#### Biceps femoris

The BF muscle responds to the hip extension in the stance phase and the knee flexion in the swing phase. By comparing the BF transfer function with the foot contact information, as shown in Fig. 2, the muscle activity can be identified as following the ipsilateral HS for the hip extension and ipsilateral TO for the knee flexion (Macleod et al. 2014).

#### Lateral gastrocnemius

The LG muscle is primarily responsible for ankle plantarflexion but also takes a minor role in knee extension (Drake et al. 2014). A peak is observed during the late stance phase when the LG muscle shortens to plantarflex the ankle, Fig. 2. The ankle plantarflexion has function to smooth the transition from double support to the swing phase (Mochon and McMahon 1980). It should be noted that only the ankle plantarflexion is considered as the primary muscle function in our study. Another peak is observed during the early stance phase, which is caused by the muscle lengthening while the hip extends forwards. This LG transfer function is excluded because the eccentric muscle contraction cannot be directly related to a dynamic movement.

#### Tibialis anterior

The TA muscle has two distinct roles during human walking: (1) to dorsiflex the ankle during the swing phase for foot clearance and placement; (2) to contract during ankle plantarflexion at the initial foot contact with the ground. It was ascertained in Fig. 2 that the muscle has a peak activity during the early stance to generate force to lower the foot, where the muscle works as a reverse muscle (Hamilton et al. 2012). Another peak in TA activity is closely related to the ipsilateral TO, corresponding to ankle dorsiflexion during the swing phase.

#### Extract transfer functions

The motor actions were subsequently related to the functional roles of each muscle. The joint movements are activated and inhibited by sensory feedback as shown in Fig. 2. The eliciting and inhibiting sensory signals of muscle activations are summarised in Table 1. The transfer functions for joint movements are derived following Eq. 5.

5 $$\begin{aligned} \begin{aligned}&H_{m,a}(t)=&{\left\{ \begin{array}{ll} H_{m,i}(t+t_{s}) &{} \quad { 0 \le t \le (t_{e}-t_{s})} \\ 0 &{} \quad \text {otherwise} \end{array}\right. } \end{aligned} \end{aligned}$$

where $t_s$ and $t_e$ are the identifiable start and end timings associated with the sensory input and joint movement. *a* indicates the corresponding joint movement where *a* = hip flexion (HF), hip extension (HE), knee flexion (KF), knee extension (KE), ankle plantarflexion (AP), ankle dorsiflexion (AD).

**Table 1:** A summary of $t_s$ and $t_e$ of transfer functions related to muscle-joint functions

Muscle	Joint function	Sensory input	$t_s$	$t_e$
TA	AP	IT	HS	HO
	AD	IH	TO	HS
LG	AP	IH	HO	TO
RF	HF	IH	TO	AEA
	KE	CH	AEA	HO
BF	HE	IH	HS	HO
	KF	CH	TO	AEA

*AP* ankle plantarflexion, *AD* ankle dorsiflexion, *HF* hip flexion, *HE* hip extension, *KF* knee flexion, *KE* knee extension *IH* ipsilateral heel, *IT* ipsilateral toe, *CH* contralateral heel *HS* heel strke, *HO* heel off, *TO* toe off, *AEA* anterior extreme angle

### Filter function optimisation

The muscle’s response to an activation signal has a characteristic shape which closely matches the impulse time response curve of a damped, linear, second-order differential system (Milner-Brown et al. 1973). In the previous study the muscle transfer functions were optimised using a curve fitting process to remove spurious artefacts and resampled at a specific sampling frequency to fit the mechanical system of the RunBot II (Macleod et al. 2014). Conversely, in this paper, a second-order low-pass Bessel filter; see Eq. 6, was used to optimise the muscle transfer functions,

6 $$\begin{aligned} \widehat{H}(t)= g\left( \dfrac{1}{\tau }\mathrm{e}^{\dfrac{-1.5 t}{\tau }} \sin \left( \dfrac{\sqrt{3}t}{2\tau }\right) \right) \end{aligned}$$

where *g* is the gain parameter to normalise the amplitude and $\tau$ is the time constant of a second-order low-pass Bessel filter, $\tau = \dfrac{1}{2 \pi f_c}$. This approach was feasible as the second-order model behaves like a low-pass filter that produces a delay between the neuronal excitation and the active state of the muscle (Reeve and Webb 2003).

**Fig. 3:**
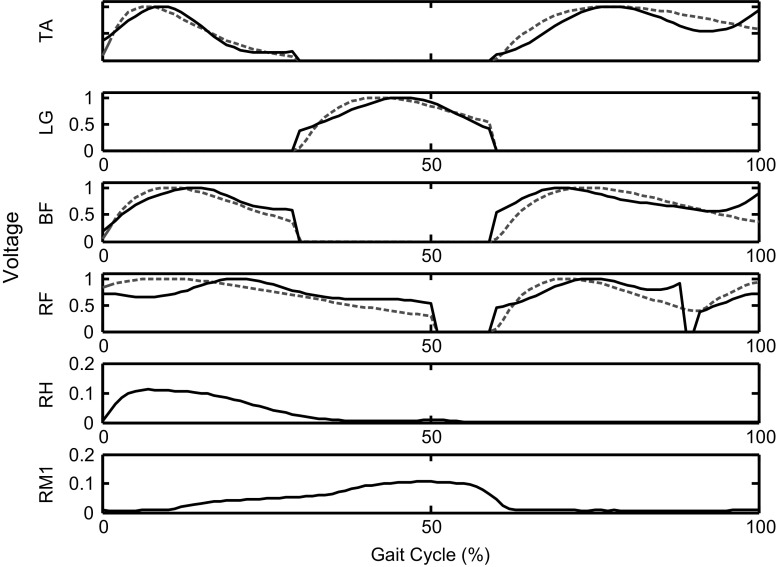
Plots of the filter functions related to one stride. Each impulse response of the filter function (red dashed line) curve fits the corresponding average muscle transfer function from all subjects (black solid line)

The impulse response of the filter function was used to effectively curve fit the desired characteristics of the average muscle transfer function from the population using the least-square error method by adjusting the cut-off frequency $f_c$. The resulting transfer functions $\widehat{H}$ were normalised to a value range between 0 and 1 with the gain coefficient *g*. The transfer functions in the form of an IIR filter could easily be adapted for the RunBot. The transfer functions created correspond to one gait cycle in duration, which is defined as the time interval between two successive foot contacts. The results of the curve fitting process are provided in Fig. 3 and Table 2.

**Table 2:** Filter functions for each joint

Muscle	Filter function	$\tau$	*g*	Sum of squared error (SSR)
TA	$\widehat{H}_{A,P_{HS}}$	0.11	15.75	0.26
	$\widehat{H}_{A,D}$	0.29	41.41	1.07
LG	$\widehat{H}_{A,P_{HO}}$	0.20	28.47	0.37
BF	$\widehat{H}_{H,E}$	0.17	23.98	0.93
	$\widehat{H}_{K,F}$	0.23	32.54	1.60
RF	$\widehat{H}_{H,F}$	0.18	25.32	0.20
	$\widehat{H}_{K,E}$	0.32	45.53	2.17

Filter functions for each joint. The filter function was used to curve fit the characteristics of the muscle functions in the normalised gait cycle

In summary, we obtained the transfer functions that relate sensory inputs to joint movement outputs. An abstract closed-loop robotic model is proposed in the next section based on these functions.

## Robotic model

### Mechanical design of the RunBot III

The RunBot III has a height of 0.3 m from foot to hip joint axis and a total weight of 552 g. It has two legs, two feet, and a small torso body attached to a boom for constricting its walking path to a planar circle. The robot consists of six actuated joints: two hip joints, two knee joints, and two ankle joints. The hips and ankles are directly actuated by DC servo motors HS-625MG (Hitec RCD, USA) and HS-85+MG (Hitec RCD, USA), respectively. The compliant knees are actuated by DC servo motors HS-85+MG (Hitec RCD, USA) via springs (ENTEX STOCK SPRINGS, UK). All built-in pulse width modulation circuits are disconnected and control voltages are applied directly to the motors. The motor positions are measured via potentiometers. The output voltages are sent to a computer running Linux through D/A acquisition devices (USB-DUX, Incite Technology Ltd, UK). The boom can rotate freely in all three axes (pitch, roll, and yaw). A summary of the robot is detailed in Table 3.

**Table 3:** Specification of RunBot III

Parameters	Value
Mass (g)	552
Dimension of thigh (cm)	4 $\times$ 0.2 $\times$ 11
Dimension of shank (cm)	4 $\times$ 0.2 $\times$ 10
Dimension of foot (cm)	6 $\times$ 1$\times$ 1
Total height (cm)	30

**Fig. 4:**
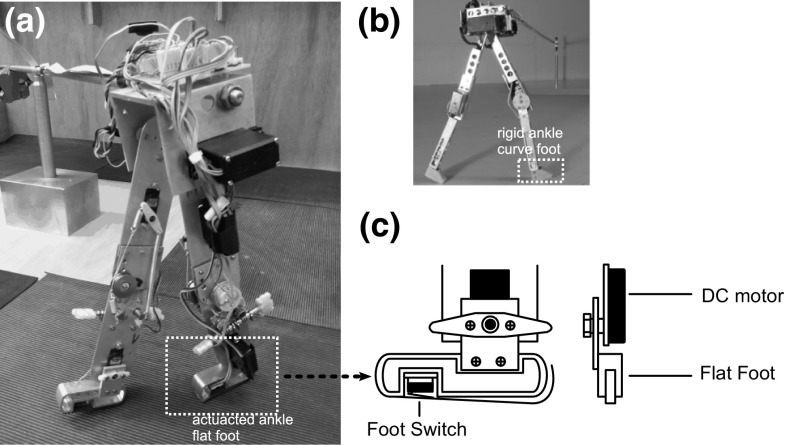
Mechanical design of the RunBot III. **a** RunBot III with the actuated ankle joint. **b** The original RunBot with the rigid ankle joint (Geng et al. 2006). **c** Ankle-foot design

The most significant change to previous versions in the mechanical design of our robot is the ankle-foot segment. The curved feet with rigid ankles used in previous generations (Fig. 4b) were replaced by flat feet with actuated ankle joints, as seen in Fig. 4c. A microswitch sensor (Maplin, UK) is placed in the foot to detect the foot contact with the ground. The foot surface is supplemented with a rubber pad with high friction and appropriate shock absorbing capability.

The RunBot III was used to validate our learn-from-human approach with regard to developing human-like walking with similar key characteristics such as joint kinematics. The ankle was of particular interest in the controller as the ankle has been identified as a major power generator in human walking (Winter 1983b). Its influence in human walking has been investigated in numerous studies (Winter 1983b; Sutherland et al. 1980; Neptune et al. 2001; Nadeau et al. 1999). Thus the functional impact of ankle movement in the RunBot III was one of our main concerns in this paper.

### Sensory feedback from ground contact

**Fig. 5:**
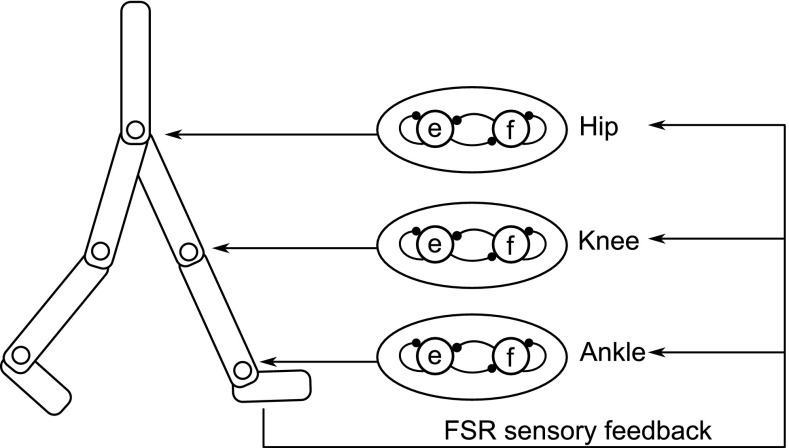
Each joint has a flexor and extensor which inhibits each other. Sensory feedback from foot contact information is sent back to the reflex generator

The closed-loop interaction between a neural controller and the biomechanical system is created by implementing our black box controller in the RunBot III, see Fig. 5. In our model the generation of walking depends primarily on the ground contact information. Each leg comprises of a hip, knee and ankle with a flexor and extensor mechanism in each joint. The flexor/extensor are elicited by afferent sensory inputs from the feet and utilise reciprocal inhibition. The three joints are coupled to generate a stable limit cycle walking.

The loading and unloading of the leg generates the impulses which elicit the reflexes according to:

7 $$\begin{aligned} \begin{aligned}&G = {\left\{ \begin{array}{ll} 1 \quad {F < \theta _{F} } \\ 0 \quad \text {otherwise} \end{array}\right. } \\ \end{aligned} \end{aligned}$$

where *F* is a real-time voltage signal from a microswitch sensor in the foot^2^ and $\theta _F$ is the threshold to define the ground contact statue *G* (1 = foot contact, 0 = foot off). $\Theta (G')$ is a positive impulse signal from the derived ground contact sensory input *G* and $\Theta (-G')$ is an impulse signal indicating that the foot is lifted off the ground.

### Motor output generation

The controller has a hierarchical structure with three loop controls: leg control, intra-joint control and local joint control. The sensory input from the foot excites the extensors of the ipsilateral leg and flexors of the contralateral leg, so-called leg control. In intra-leg control, when the hip achieves its AEA during the swing phase, the AEA signal will activate an extensor reflex at the ipsilateral knee. The local joint reflex arises in joint control to inhibit the motor output to prevent the hyperflexion or hyperextension of the joint.

The total motor outputs are defined by $U_{H/K/A, F/E/P/D}$ and generated by convolving the summation of sensory feedback signals with the corresponding transfer functions, Eq 8:

8 $$\begin{aligned} U_{H, F}= & {} ~B_{H,F}\widehat{H}_{H,F} *(\omega _{H,F} \Theta (G_C')) \nonumber \\ U_{H, E}= & {} ~B_{H,E}\widehat{H}_{H,E} *(\omega _{H,E} \Theta (G_I')) \nonumber \\ U_{K,F}= & {} ~B_{K,F} \widehat{H}_{K,F} *(\omega _{K,F} \Theta (G_C')) \nonumber \\ U_{K,E}= & {} ~B_{K,E} \widehat{H}_{K,E}*(\omega _{K,E}\Theta (-\dot{B}_{I,H, F})) \nonumber \\ U_{A,P}= & {} ~U_{A, P_{HS}} + U_{A, P_{HO}} \nonumber \\= & {} ~B_{A, P_{HS}} \widehat{H}_{A,P_{HS}} *(\omega _{A, P_{HS}} \Theta (G_I')) \nonumber \\&+B_{A, P_{HO}} \widehat{H}_{A,P_{HO}} *(\omega _{A, P_{HO}}\Theta (-G_I')) \nonumber \\ U_{A,D}= & {} ~B_{A,D} \widehat{H}_{A,D} *(\omega _{A, D} \Theta (G_C')) \end{aligned}$$

where *G* is the ground contact signal with *I* defining the ipsilateral leg and *C* presenting the contralateral leg, and $\omega$ the weights of the connections between the sensory inputs and motor outputs. *B* are signals from the stretch receptors that inhibit the motor outputs when the joints flexing or extending beyond an extreme angle threshold. $\Theta (-\dot{B}_{I,H, F})$ defining the moment that the ipsilateral hip achieves the AEA during the swing phase is used as an impulse trigger signal to elite knee extension of the ipsilateral leg.

Transfer functions $\widehat{H}$ translate sensory impulse signals into motor neural activations. They were modelled by a time parameter $\tau$ and a gain coefficient *g*, equivalent to human transfer function (Eq. 6). Because the coefficients $\tau$ derived from human data (Table 1) were determined in a normalised gait cycle, they were multiplied with predicted stride time before being applied to the robotic control as initial values. The $\tau$ values in Eq. 8 were carefully tuned.

### Reflex to robotic control

The flexor and extensor reflexes were used to generate the motor command to drive the corresponding joint actuator. The voltage of the joint motor is obtained similarly to that presented in the reflexive model discussed in Geng et al. (2006); Manoonpong et al. (2007):

9 $$\begin{aligned} \begin{aligned} V_H&= ~s_H \cdot \alpha _H \cdot (U_{H,F} - U_{H,E})\\ V_K&= ~s_K \cdot \alpha _K\cdot (U_{K,F} - U_{K,E}) \\ V_A&= ~s_A \cdot \alpha _A \cdot (U_{A,F} - U_{A,E}) \\ \end{aligned} \end{aligned}$$

where *V* is the input voltage of the motor, $\alpha$ represents a servo amplifier coefficient and $U_E$ and $U_F$ are the outputs of extensor and flexor motor neurons. *s* is $+1$ or $-1$, which indicates the signs of the motor voltages of flexion and extension in the joint, depending on the polarity of the motor.

The integration of the reflex outputs were mapped to the biped robot, which is actuated by a single DC motor for each joint.

## Experimental results

The next stage was to apply the reflexive control model to the RunBot III and to analyse the resultant gait. The parameters, such like servo amplifier coefficients of motors $\alpha$ and load receptor thresholds $\theta$, were determined in order to achieve a stable walking pattern. The optimised choice was selected based on the stability and the walking speed by trials and errors (Table 4).

**Table 4:** Optimal parameters for the RunBot III

		$\widehat{H}$			$\theta$ (Deg)	$\alpha$
		$\tau$	*g*	$\omega$		
L/R						
Hip	*F*	0.23	85.89	1	120/100	1.5
	*E*	0.23	85.89	1	75/65	
Knee	*F*	0.13	37.94	1	90/90	3
	*E*	0.13	37.94	1	0/0	
Ankle	$P_{HS}$	0.08	28.46	0.75	$-10$/$-10$	2
	$P_{HO}$	0.13	37.94	0.75	$-15$/$-15$	
	*D*	0.05	18.99	1	15/15	

**Fig. 6:**
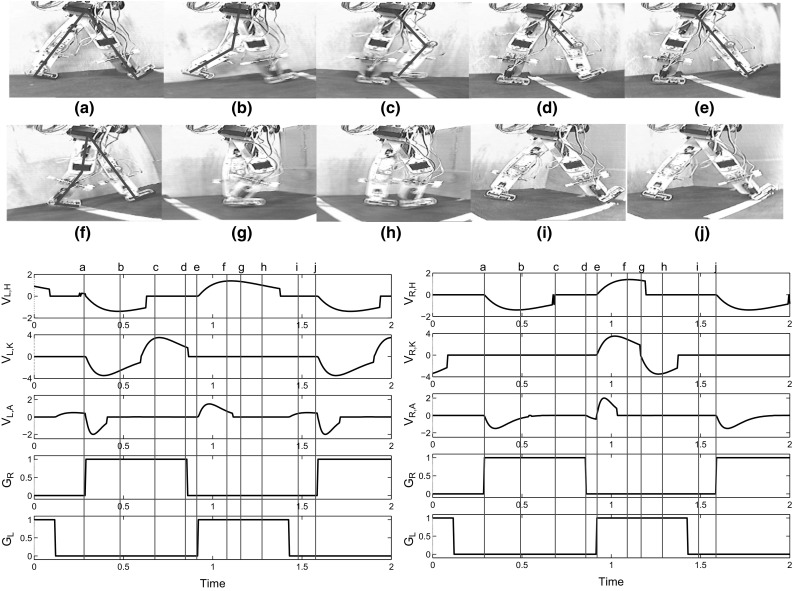
One stride of the RunBot III. Top: frames captured from a video file of the robot walking. Bottom: the control voltages of the right leg and left leg and the ground contact information (GL and GR). **a** Foot touch down on right. **b**, **c** The right foot contact triggers the extensors of the right leg and flexors of the left leg. **d** The plantarflexion on the right in response to the heel off. **e** Ground contact on the left. **f**, **g** The right leg initiates the swing phase while the left leg is in the stance phase. **h** When the right hip reaches its AEA, the right knee starts to extend in (**i**). **j** The right foot contacts the ground again and one stride finishes

### Rhythmic behaviour: walking in a circular path

Video frames demonstrating one stride of RunBot III are shown in Fig. 6 (top). Only the control of right leg (red) is described here as the control model is symmetric, Fig. 5.

At (a), the right foot touches the ground which initiates the stance phase of the ipsilateral leg The extensors of the right leg are activated. At (b) and (c), the right hip and knee continue to extend and the foot rotates towards to the ground so that the whole leg rotates forward like an inverted pendulum. At (d), the lift-off of the right foot activates the ankle plantarflexion in late stance phase. At (e), the left foot contacts the ground. The two legs switch their swing and stance roles. The left foot contact signal excites the swing phase of the right leg and the flexors of the right leg are activated. At (f), the right hip flexes forwards while the right knee and ankle flexes to clear the foot from the ground in early swing phase. At (g), the right hip reaches its AEA which causes the inhibition of the knee flexor and excitation of the knee extensor. At (h) and (i), the right knee continues to extend until the leg is straight. And finally at (j), the right foot contacts the ground and the gait cycle returns to (a).

### The relationship between the hip extension and ankle push-off

To examine the coupling effect between the hip extensor velocity and ankle plantar flexor velocity after HO and its impact on the walking performance of the robot, the RunBot III was driven by varying the servo amplifier coefficient of the hip $\alpha _H$ (from 1.3 and 1.6 with a step of 0.1) and the weight parameter $\omega _{A, P_{HO}}$ (from 0 to 1.25 with a step of 0.25).

**Fig. 7:**
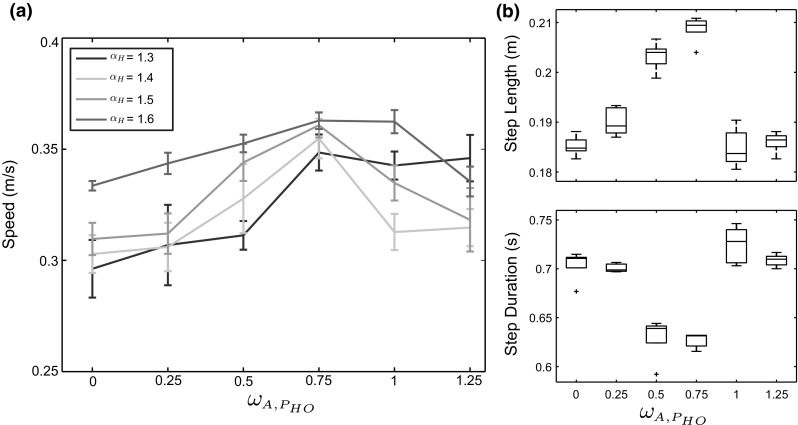
**a** Speed results as a function of the weight of the ankle plantarflexor $\omega _{A, P_{HO}}$ and the servo amplifier coefficient of the hip $\alpha _{H}$. **b** Box plots comparing RunBot’s step length and step stride using various weight values of $\omega _{A, P_{HO}}$ when the $\alpha _{H}$ is set to 1.5

Figure 7a describes how the walking speed performance of RunBot III responds to all coupling combinations of these two parameters. The robotic walking speed increases with the addition of ankle push-off. When the weight $\omega _{A, P_{HO}}$ increases from 0, a larger impulsive ankle push-off contributes to a shorter step duration and a larger step length (Fig. 7b) resulting in a faster walking speed. Moreover, a maximal speed was obtained when the weight $\omega _{A, P_{HO}}$ reached 0.75. An excessive ankle push-off leads to a relatively slow speed as it impedes on the natural walking dynamics of the robot, producing shorter step length and longer step time, see Fig. 7.

**Fig. 8:**
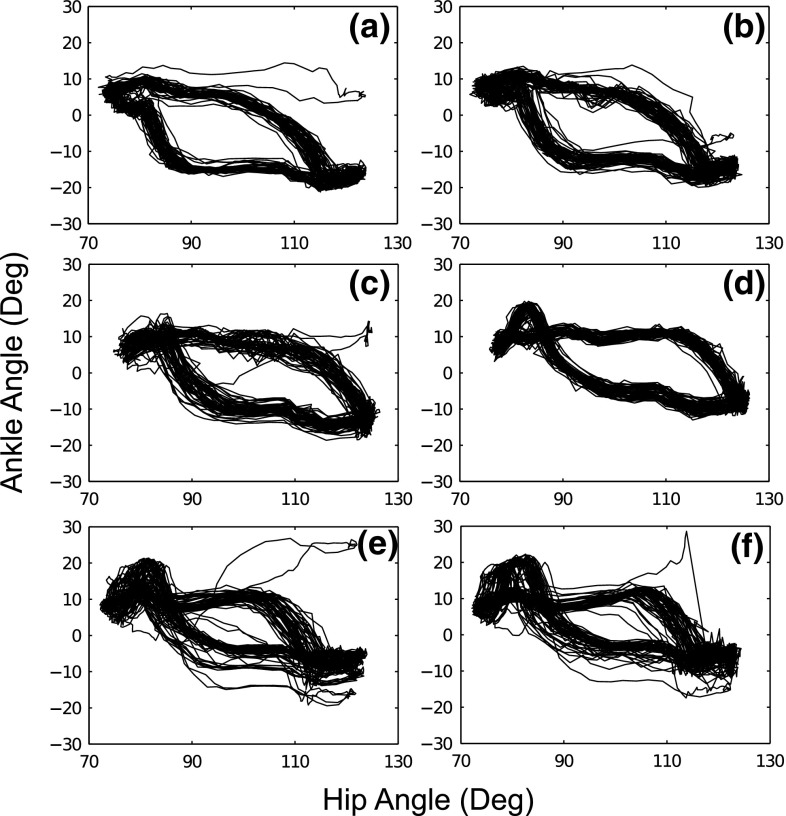
The plots of ankle angular motion versus hip angular motion with various values of $\omega _{A, ~P_{HO}}$ during the gait cycle. **a** $\omega _{A, ~P_{HO}} = 0$. **b** $\omega _{A, ~P_{HO}} = 0.25$. **c** $\omega _{A, ~P_{HO}} = 0.5$. **d** $\omega _{A, ~P_{HO}} = 0.75$. **e** $\omega _{A, ~P_{HO}} = 1$. **f** $\omega _{A, ~P_{HO}} = 1.25$. Note here $\alpha _{H} = 1.5$

A coupling effect between the hip extensor velocity and ankle plantarflexor velocity at late stance was observed in Fig. 7a. The robot was able to perform a faster walking speed with a lower hip velocity and a higher ankle push-off velocity compared to the robot’s walking speed with a higher hip velocity and a lower ankle push-off velocity. For instance, a faster walking speed of 0.3484 ± 0.0081 m/s (mean ± SD) was obtained when $\omega _{A, P_{HO}}$ and $\alpha _{H}$ were respectively set to 0.75 and 1.3 compared to a walking speed of 0.3118 ± 0.0087 m/s when $\omega _{A, P_{HO}}$ was 0.25 and $\alpha _{H}$ was 1.5. The results demonstrate that there is a trade-off between the ankle push-off velocity and the hip extensor velocity with regards to the walking speed performance of the robot.

### Stability analysis

We investigated the stability of the limit cycle walking model. In regard to the dynamic interaction between the hip and the ankle joints, the phase diagrams of the ankle angular motion with the hip angular motion during a walking period of 100 steps, with an augmented ankle push-off velocity, were shown in Fig. 8.

Starting from the initial position, these diagrams show the convergence of the walking cycle into a limit cycle. Although the gait stability is affected by varying values of ankle push-off velocity $\omega _{A, P_{HO}}$, we can see that overall the control system produces stable limit cycles. The RunBot III produced the fastest walking speed when $\omega _{A, P_{HO}}$ was set to 0.75. The corresponding phase plot (Fig. 7d) demonstrated that the limit cycles were significantly less affected by perturbations than the other settings and so appear more stable. This suggests that the ankle push-off velocity significantly affects the stability of the walking system because of a significant amount of energy generated from the ankle joint during “push-off". limit cycles appear less stable when the weight value was set to > 0.75 as shown in Fig. 8e and f. Excessive energy injection into the mechanical system may increase the time for the biped walker to converge to a dynamic stable condition and result in a slower walking speed (Fig. 7).

**Fig. 9:**
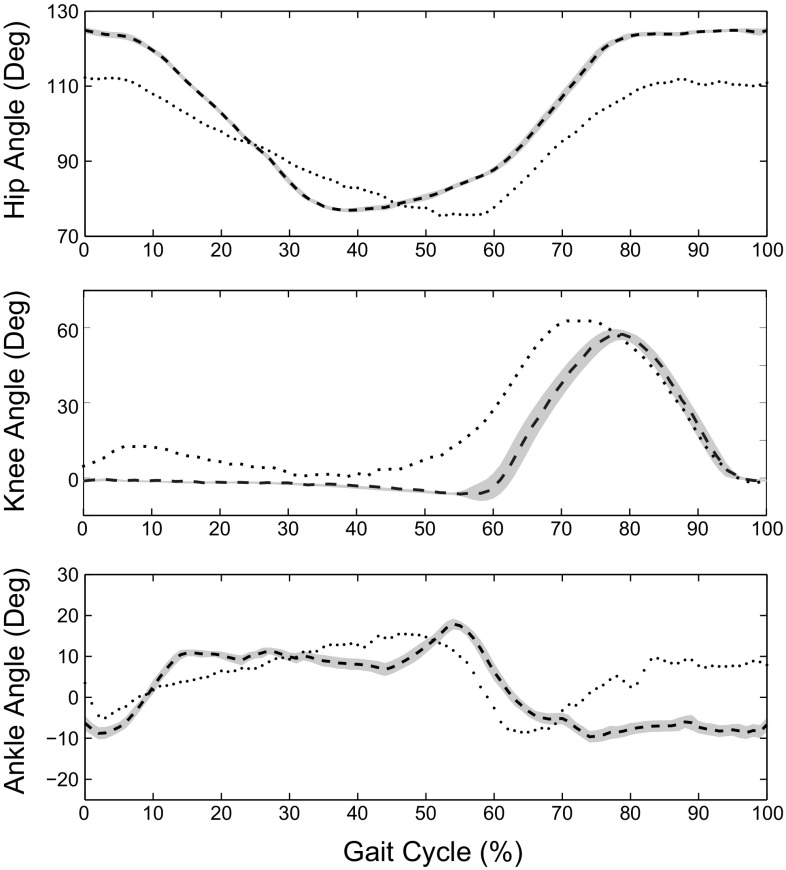
Kinematic comparison between the robot and human joint angles during one gait cycle. The averages (dashed line) with standard deviation (gray shaded) across one gait cycle of the robot and human motion (dotted line) are shown in one plot

### Comparison to human

**Fig. 10:**
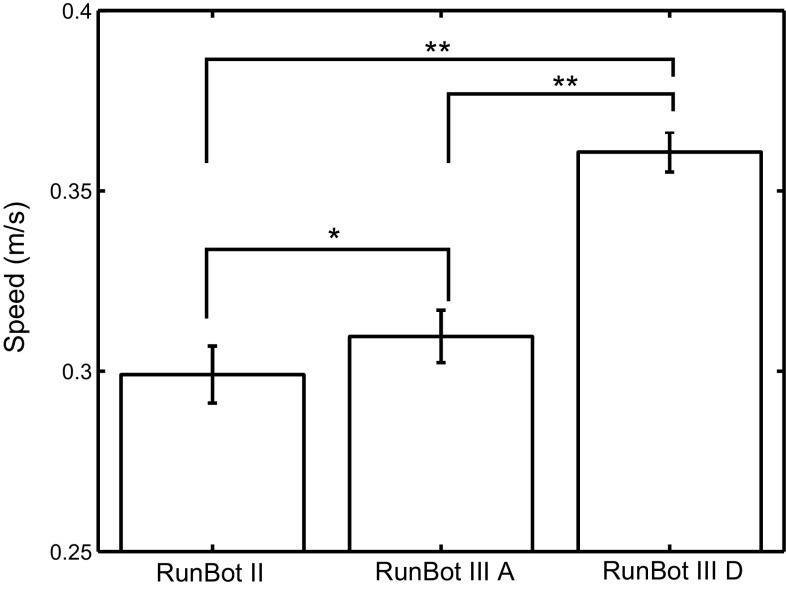
Speed comparison between the RunBot II and RunBot III without ankle push-off ($$\omega _{A, P_{HO}} = 0$$) and with optimal ankle push-off ($$\omega _{A, P_{HO}} = 0.75$$). where $$*~p < 0.05$$. $$**~p < 0.001$$

Robotic behaviour can be used to provide insight into the biological mechanism of human walking. It is thus of interest to compare the measured results from the RunBot III to human gait data:

1. The RunBot III achieved a similar relative walking speed to humans. The relative walking speed is defined as a speed corrected for leg length. The maximal relative walking speed of the robot is 1.2 leg length/s compared to the approximate relative walking speed of 1.45 leg length/s where a human subject with an average height of 1.75 m walks at a preferred speed of 1.4 m/s (Browning et al. 2006).
2. The RunBot III attained a close efficiency of walking to that of human with the addition of actuated ankle control. In the study of JudgeRoy et al. (1996), a ratio of 0.74 between step length and leg length used to represent a human subject had an efficient ankle plantarflexor during the late stance phase. The robot had a step length of 0.7/leg length when $\omega _{A, P_{HO}}$ and $\alpha _{H}$ were, respectively, set to 0.75 and 1.5 (see Fig. 7b), which shows a good approximation to human walking. The addition of ankle movement significantly contributes to the walking speed of the RunBot III with a 16$\%$ increase in speed compared to the RunBot II with a rigid ankle (Macleod et al. 2014) (Fig. 10).
3. The robot joint motion quantitatively matched the human data in literature [literature human data derived from van der Linde (1999) as shown in Fig. 9]. Characteristics and timing were similar while amplitude were slightly different. Some differences were observed due to limitations of the robotic design. The robot lacks the knee flexion in the stance phase due to a mechanical stop in the knee joint. In addition, because the isometric muscle contractions during stance phase were not considered in the model, the ankle joint was passively driven with the leg inverse rotation after full ground contact, and therefore a slightly passive dorsiflexion was observed during mid-stance.

## Discussion

Our approach takes inspiration from the pattern of sensorimotor coordination in humans (Taga 1995). The development of a reflexive control system based on filter functions derived from human walking data aimed to demonstrate that using simple sensory feedback can be a successful method to adaptively coordinate the limb segment movements and generate a stable walking in a robotic walker. We have shown that there is a direct causal relationship between foot contact information and muscle activity during human walking in our previous study (Macleod et al. 2014). The causal relationship allowed us to generate a reflexive robotic system, which reproduces the activations of the relevant muscles after foot contact. Our reflexive controller exploits the natural dynamics of the RunBot III for locomotion generation without the requirement of CPGs or trajectory control.

Walking can be considered as a nominally periodic sequence of steps which although not locally stable at every instant time, is stable at a whole, so-called limit cycle walking (Hobbelen and Wisse 2008). This allows a robot to adapt its gait to changing natural dynamics producing a convergence to a desired motion using low or no feedback gains (Collins and Ruina 2005; Wisse 2005; Wisse and Van Frankenhuyzen 2006). Most limit cycle walkers utilise an approach where an oscillator controller, e.g. CPGs, and mechanical systems are dynamically coupled to generate stable limit cycle stepping. This approach was promising in solving robot locomotion control as it aimed “to achieve a robust and adaptive behaviour while coordinating a redundant high degree of freedom system under the strong effect of physical body dynamics” (Miyakoshi et al. 1998). This is more efficient than using high feedback gain to constrain the robot walking on an intended path. Our controller demonstrates that limit cycle walking in RunBot is able to return naturally to the desired trajectories following a disturbance without CPGs or trajectory tracking.

Local circuits in the spinal cord have been identified as being responsible for locomotor movements in both vertebrates and invertebrates (Brown 1911; Grillner 1985; Rossignol 2000). Studies have revealed that in various animal species locomotion is driven by CPGs, which generate the rhythmic motor outputs without sensory or descending inputs carrying specific timing information (Marder and Bucher 2001; Ijspeert 2008). CPGs have been identified in mammals such as the cat but their existence in humans has not been conclusively described (Ijspeert 2008). The difference between humans and other species can be observed following a complete spinal cord injury, humans become completely paralysed below the injury, whereas rhythmic stepping can be evoked in a cat after complete spinal transaction. This demonstrates that human locomotion control is more dependent on intact supraspinal control than is found in the cat (Hultborn and Nielsen 2007). Unlike a CPG, a reflex is a local motor response to a local sensory input. In human locomotion, a chain of reflexes act together to control the limbs and their integration contributes to the regulation of the locomotion pattern (Zehr and Stein 1999). Reflexes are dependent on task, phase, and context and therefore require modulation using sensory feedback from peripheral afferents in order to contribute effectively in locomotion, where the initial conditions may change on every step.

Most bio-inspired robotic models have been built based on CPGs generating basic components of rhythmic motor patterns (Lewis et al. 2005; Iida et al 2008; Klein 2011). Sensory feedback has been used to regulate rhythmic activities of the neural controller, compose coupled neural oscillators and coordinate relevant movements of a neural-mechanical system. However, the interaction between the nervous system and mechanical system has often been modelled based on neuronal processing algorithms, resulting in complicated models with a high computation requirement.

This study emphasised the potential of a human-inspired framework in the design of a locomotion controller, which utilises human data and output functions that appear to be intrinsic to human walking. The neural-mechanical interaction can be regarded as a black box where the muscles serve as actuators and the limbs are regarded as linkages (Pandy 2001). Prentice et al. (1998) developed a neural network model that replicated the role of CPGs in human locomotion, based on gait cycle and EMG data recorded from one participant walking on a treadmill. The model was limited in its representation of certain aspects of the EMG profiles due to lack of sensory feedback. Foot contact can be used alongside EMG for analysis of muscle function as it provides spatial and temporal information during locomotion. Different strategies for generating control based on muscle activity and foot contact have been studied for use in human motor control or rehabilitation (review in Sinkjaer et al. 2003). However, to the authors’ knowledge, the transfer functions which directly relate foot contact and muscle activity derived from human data have not been employed to create a minimal closed-loop controller based on the coupling between sensory inputs and motor outputs.

Human leg mechanics can be encoded into autonomous muscle reflexes (Geyer and Herr 2010). The variation in EMG measurement is an important concern as natural variation of leg length, muscle size, stride length, and other physiological factors will influence muscle activity during walking. During data acquisition we aimed to control variation due to walking speed as much as possible and the differences between men and women were also considered. Further details and an analysis of variation in transfer functions derived from different groups was discussed in our previous paper (Macleod et al. 2014). Filter functions were used to optimise the EMG transfer functions as the muscle response forms a characteristic shape which closely matches the impulse time curve of a damped, linear second-order differential system (Reeve and Webb 2003). The time constant $\tau$ describes the response time ($t_r = 0.6068 \tau$) of a burst activity from the excitation moment to reaching its maximal amplitude instead of the duration of transfer functions as the $\tau$ determines the characteristic shape of transfer function. The $\tau$ derived from human data (Table 1) based on the gait cycle ($0 \sim 100 \%$) will be easily adapted to walking speeds by multiplying with the stride time. The use of IIR filter functions allowed us to increase the adaptability and efficiency of our control system. Although the parameters in RunBot III were determined by trial and error with the aim of obtaining an optimal speed, an adaptive control of the robotic speed is of significant interest and will be a subject of further research.

Ankle push-off during late stance in human walking has been described as having an important role in facilitating the initiation of the swing phase (Neptune et al. 2001; Renjewski and Seyfarth 2012; Lipfert et al. 2014). The results shown in Fig. 7 demonstrate that the ankle push-off contributes greatly to the walking speed of the RunBot III. The $\omega _{A, P_{HO}}$ equal to 0 means that no ankle plantarflexion occurs during the late stance phase. The increasing weight parameter $\omega _{A, P_{HO}}$ augments positive power generated in the ankle joint, resulting in increasing the walking speed. It should be noted that the ejection power may produce a disturbance to the natural dynamics and a decrease of speed if the $\omega _{A, P_{HO}}$ reaches its threshold (Fig. 8) as the work may cause redirection of the centre of mass (Lipfert et al. 2014). The neural controller and mechanical system was dynamically coupled to generate a limit cycle walking in the robotic system, which allows the RunBot III to exploit its natural dynamics following a disturbance and converge back to a stable gait without any trajectory control.

RunBot is driven by local reflexes without any trajectory tracking algorithms or CPGs (Geng et al. 2006; Manoonpong et al. 2007). Phase switching of the legs is triggered by ground contact signals. When one leg contacts the ground, the signal triggers motors driving hip flexion/extension and knee flexion/extension of the swing/stance legs. The original RunBot attempted an approach to generate motor signals using a biologically inspired neuronal processing model. However, a control system comprised of neural networks is highly speculative as the human nervous system is complex and has numerous unknown variables. In our previous study, we proved the hypothesis that transfer functions derived from human data could be implemented in the reflexive controller (Macleod et al. 2014). The relationship between the sensory input and motor output can be regarded as a black box and calculated by relating foot contact information and muscle EMG signals recorded during human walking. In this paper, the ankle control was initially implemented in the reflexive controller based on the causal relationship between the foot contact information and muscle EMG activity. The HO signal is used to activate the ankle push-off while the ground contact signal triggers the ankle plantarflexor of the stance leg and dorsiflexor of the swing leg.

We presented a human-inspired approach to bipedal robotic walking by using only human data and the causal relationship between the sensory feedback and motor outputs. To our knowledge, this is the first attempt to extract biological reflex principles from human gait studies for controlling robotic locomotion. The addition of ankle control was initially considered within the reflexive controller to advance our understanding of the role of the ankle in developing a functional and efficient gait. The RunBot III was subsequently constructed and used to validate the control principle. Stable walking in the RunBot III demonstrated that the generated limit cycle was able to return naturally to the desired trajectory following a disturbance after only a short time without the addition of CPGs or trajectory control. The practical application of the strategy presented in this paper indicate a promising future of a “human-inspired” gait control approach utilised in robotic locomotion controller design.

## Electronic supplementary material

Below is the link to the electronic supplementary material.

Supplementary material 1 (mp4 37280 KB)

## Footnotes

1 The heel and toe contact information in humans are continuous analogue signals, while foot contact switches (ON/OFF) were implemented in the RunBot III.

2 Although it has been observed that toe off is an essential event for indicating the start of the swing phase, RunBot III does not feature a toe contact sensor and the foot was designed as a whole rigid body. Instead, the contralateral foot contact was used as the trigger of the swing phase of the leg as in previous RunBot iterations. The foot contact detected by the foot switch as shown in Fig. 4c concurs with heel strike in human walking while the foot off indicates the heel off event.
